# Comparative in vitro study on wear resistance and colour stability of 3D-printed, milled, and conventional PMMA denture teeth

**DOI:** 10.1186/s12903-025-05983-7

**Published:** 2025-04-29

**Authors:** Yasmine Mahmoud Mohamed Elsharkawy, Hebatallah Tarek Mohamed, Tarek Mohamed Al Sayed El Saeedi, Amany Mostafa Saad Farahat

**Affiliations:** 1 Department of Oral and Maxillofacial Prosthodontics, Faculty of Dentistry, Ain Shams University, Cairo, Egypt; 2 Department of Oral and Maxillofacial Prosthodontics, Faculty of Dentistry, Ain Shams University, Organization of African Unity St., off El-Khalifa El-Mamoun St., Abbassia, Cairo, Egypt

**Keywords:** Digital denture, Denture tooth, PMMA, Chewing simulator, Colour stability, Thermocycler, Spectrophotometer

## Abstract

**Background:**

Digital dentures are a promising alternative to the conventional fabrication technique. However, their mechanical and optical properties require further evaluation, so this study aims to compare the wear resistance and colour stability of milled and 3D-printed polymethyl methacrylate denture teeth to conventional teeth. This is essential as too much wear can cause a loss of the vertical dimension of occlusion (VDO), which can compromise the chewing efficiency, esthetics, and even cause the dentures to break.

**Methods:**

Mandibular first molars denture teeth were manufactured using three different techniques, which were chosen to be assessed for wear resistance and colour stability. In the first group, conventionally prefabricated mandibular first molars were used; in the second group, the molars were milled from PMMA blocks; in the third group, the molars were fabricated from 3D printing resin (*n* = 7). The teeth were loaded on the chewing simulator to simulate the intraoral conditions, and then the volumetric changes were evaluated using surface matching software. Teeth were subjected to aging using the thermocycler, and colour stability was evaluated using a spectrophotometer.

**Results:**

The null hypothesis was rejected, indicating significant differences between the groups. For wear resistance, the highest mean wear (RMS) value was reported in the conventional group, 1.806 ± 0.085, followed by the printed group, 0.021 ± 0.006, and then the milled group, 0.019 ± 0.005. For colour stability, the highest mean value of colour change (Delta E) was reported in the printed group 2.996 ± 0.445, followed by the conventional group 2.725 ± 0.234, and then the milled group 0.539 ± 0.118.

**Conclusion:**

Milled PMMA generally demonstrates better wear resistance and colour stability compared to 3D-printed and conventionally processed PMMA. 3D-printed PMMA exhibits comparable wear resistance to milled PMMA. 3D-printed PMMA demonstrated comparable colour stability to conventional resin.

## Background

With better life standards and improved health insurance, the number of completely edentulous patients decreases. Despite the variety of available treatment options for oral rehabilitation to restore both function and esthetics, a complete denture is one of the most convenient options for those with medical and financial concerns that eliminate the use of other options, including implants [1].

Many materials have been used in the manufacture of denture teeth. From wood to porcelain, polymethylmethacrylate (PMMA) is the most currently used material because of its reasonable cost, lightweight, good esthetics, and easy processing. Despite its good features, PMMA material has many drawbacks related to its inherent properties, such as porosity, fracture strength, polymerization shrinkage, colour stability, and biocompatibility. These challenges associated with using PMMA for tooth fabrication can be mitigated by adopting new fabrication techniques [2].

Recently, computer-aided design and computer-aided manufacturing (CAD/CAM) technology have been intensely involved in manufacturing dental prostheses, with evidence that better characteristics can be obtained than with conventional manufacturing techniques [3]. CAD/CAM manufacturing techniques encompass both subtractive (milling) and additive (3D printing). The dental subtractive technique uses the end-milling of a fixed-size solidified block such as zirconia, wax, resin, or metals. Although this technique spreads massively, it has some drawbacks, including wasting material, increased cost, instrument wear, and difficulty accessing some areas [4].

The additive technique involves building the restoration layer by layer [5]. Using photopolymerization to solidify liquid-based materials layer by layer, stereolithography (SLA) is a rather popular additive manufacturing technique. SLA 3D printing and specialized dental resins are revolutionizing denture fabrication, with numerous companies introducing innovative materials. Due to their potential benefits over prefabricated denture teeth including, being cost-effective and truly customized accurate teeth. SLA technology for 3D-printed denture teeth offers promising clinical outcomes [6]. Recently, 3D-printed complete dentures offer a promising treatment approach, yet they are considered a relatively new modality in clinical practice [7].

Tooth wear is multi-factorial process that varies according to several factors, including the consumed diet type, chewing pattern, antagonistic material, and neuromuscular force. Excessive tooth wear results in a loss of vertical dimension that impacts the normal path of jaw movement during mastication, leading to chewing difficulty and muscle strain negatively affecting the overall appearance [8].

To predict the in vivo performance of dental materials, preclinical evaluation of their wear and fatigue behavior is essential. Chewing simulators are utilized to replicate the dynamic loading conditions encountered during mastication, providing valuable data for material selection and optimization [9].

Colour stability is a crucial quality for maintaining denture teeth esthetics. Tooth discolouration impacts patient satisfaction and long-term quality of life. Various factors contribute to denture tooth colour change, such as water absorption, stain accumulation, pigment deterioration, and increased surface roughness. Colour stability evaluation for denture teeth can be performed either visually or instrumentally. Spectrophotometry is one of the most commonly used instruments that measure the spectral transmittance and reflectance of materials, providing objective data about colour stability [10].

The objective of this study is to compare the wear resistance and colour stability of conventional, milled, and 3D-printed resin used for denture tooth fabrication. The null hypothesis was that the three materials exhibited no significant differences regarding wear resistance and colour stability.

## Methods

### Study design and sample size

In this in vitro study, mandibular first molars were manufactured using three different techniques, which were chosen to be assessed for wear resistance and colour stability. Power analysis was conducted to ensure adequate statistical power to test the null hypothesis, which posits that there is no difference in wear resistance and colour stability between the three groups under investigation. By adopting an alpha (α) level of 0.05, a beta (β) level of 0.2 (i.e., power = 80%), and an effect size (f) of 0.738 calculated based on findings of a previous study [11], the predicted total sample size (n) was found to be 21 specimens (7 specimens per group). The sample size calculation was performed using G*Power version 3.1.9.7. In the first group, conventionally prefabricated mandibular first molars were used; in the second group, mandibular first molars were milled from PMMA blocks; in the third group, mandibular first molars were fabricated using 3D printing resin.

### Tooth manufacturing

For the first group, seven acrylic prefabricated mandibular first molars (Acrostone, Cairo, Egypt) were chosen and numbered from one to seven. Utilizing a desktop scanner (D850, 3Shape, Copenhagen, Denmark) of 7–8 μm accuracy and previously calibrated in conformity with the manufacturer’s specifications, each molar was scanned, and then the scans were exported as standard tessellation language (STL) files. These STL files were considered reference scans for the first group.

The STL files were imported into CAD software (Exocad, Dental CAD 3.0 Galway; Exocad GmbH, Darmstadt, Germany), where a number from one to seven was engraved on the tooth ridge lap, and the new STL files were saved to be used for the fabrication of the specimens of the second and third groups.

For the second group, the STL files were integrated into a dental milling machine of 5 axes (DWX-52DPlus, Roland DG, Tokyo). The specimens were milled from white PMMA blanks (Ivotion Dent, Ivoclar, USA). The specimens were digitized by a desktop scanner (D850, 3Shape, Copenhagen, Denmark), and the STL files were considered reference scans for the second group.

**Fig. 1:**
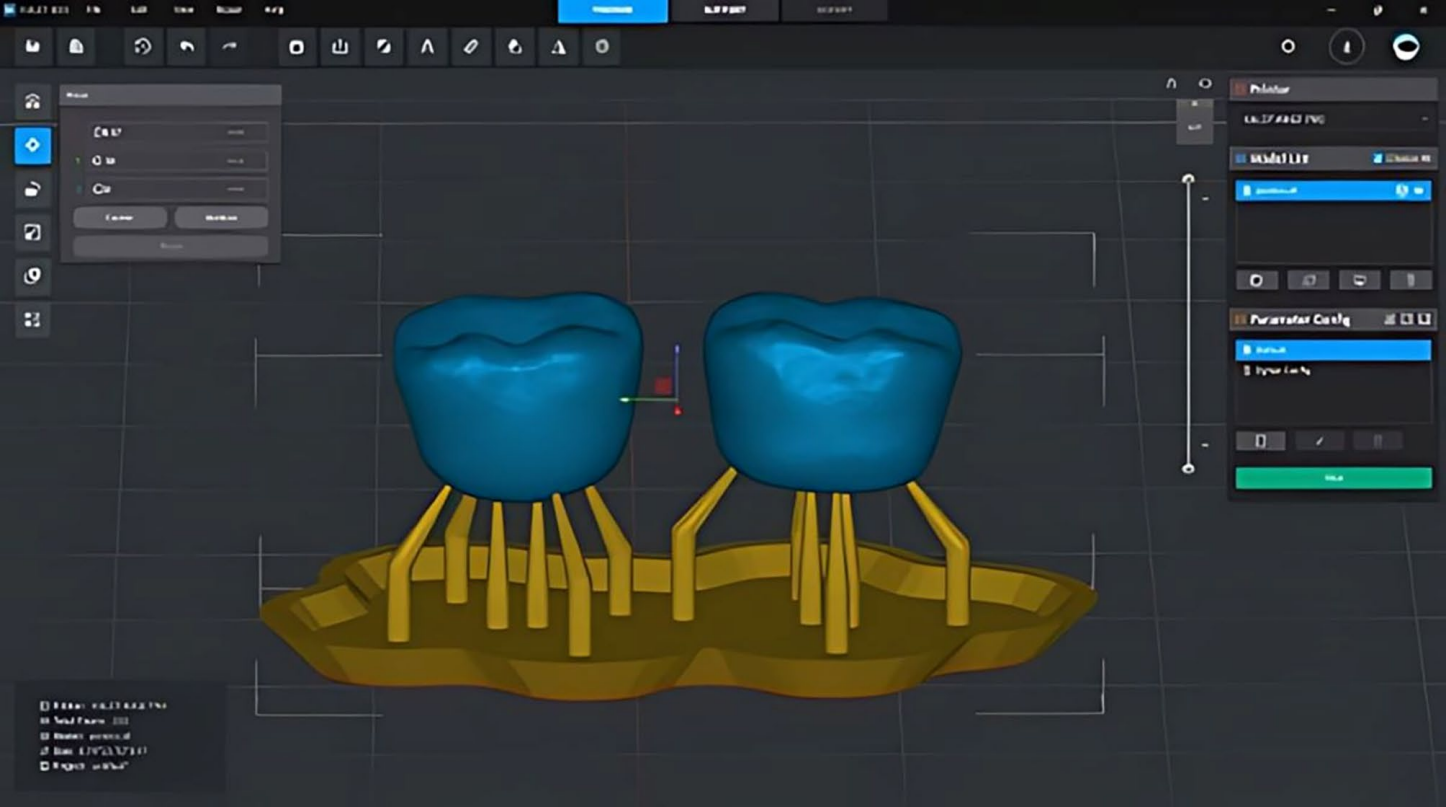
Supporting arms added to the teeth ridge lap in the 3D-printed group

For the third group, the STL files were integrated into 3D printing software (Netfabb software, Autodesk Media and Entertainment, USA) to add the supporting arms to the ridge lap of the tooth (Fig. 1), then to a digital light processing (DLP) printer (DentCase printer, Mogassam, Egypt). The specimens were printed using polymethyl methacrylate resin (NextDent CB MFH, 3D Systems, The Netherlands) with a build volume of 120 × 75 × 100 mm.

The printer and the resin were calibrated following the manufacturer’s guidelines. After printing, specimens were rinsed twice with ethyl alcohol for 3 min and air-dried for 15 min. The specimens were positioned into the post-curing unit (DentCase printer, Mogassam, Egypt) for 15 min to ensure the curing of the unreacted monomer. All the specimens were digitized with the former desktop scanner, and the STL files were saved and considered reference scans for the third group.

### Chewing simulation

**Fig. 2:**
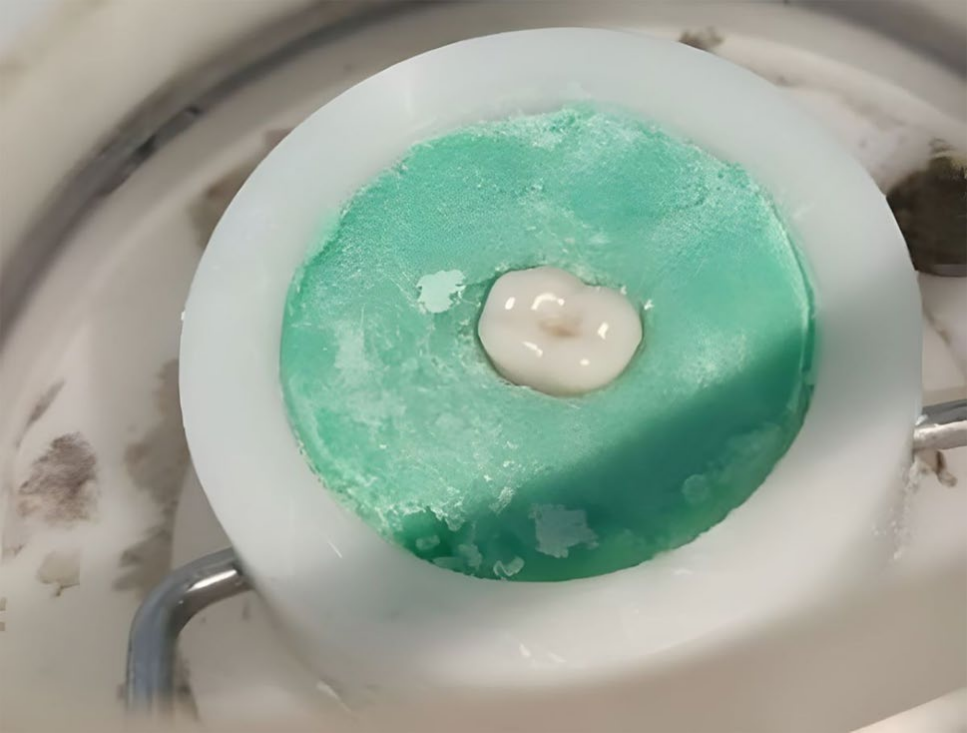
Conventional tooth specimen secured in the middle of its holder using auto-polymerizing resin

**Fig. 3:**
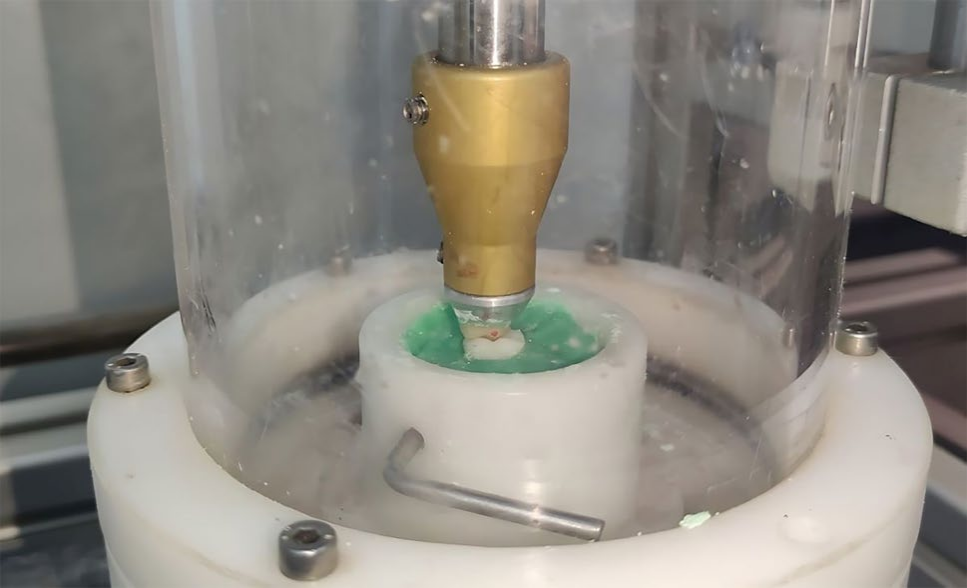
Mandibular second molar tooth in a class I relation Angle’s classification to its zirconia antagonist

Each specimen was secured in the middle of its holder using auto-polymerizing resin (Acrostone, Cairo, Egypt) to keep its position during the test (Fig. 2). The position of each tooth was standardized using a dental surveyor (Ney Tech, USA) [12]. Artificial saliva prepared according to glandosane’s formula (10.2 mmol/l NaCl, 10.7 mmol/l KCl, 0.29 mmol/l MgCl_2_.6H_2_O, 1.08 mmol/l CaCl_2_.2H_2_O, 2.20 mmol/l KH_2_PO_4_, 4.59 mmol/l K_2_HPO_4_, 2.38 mmol/l NaHCO_3_, 0.25 g/l Bio-trypticase, 0.25 g/l yeast extract, 1.01 Aqua dest.) [13] by the pharmaceutical industry laboratory at the Faculty of Pharmacy, Ain Shams University was used to cover the teeth’s occlusal surface to mimic the in vivo settings. A chewing simulator (CS4, SD Mechatronik, Feldkirchen-Westerhan, Germany) with parameters set as follows was used: weight 5 kg, equivalent to chewing force 49 N, at a frequency of 1.7 Hz (102 cycles/minute), the vertical stroke length is 2 mm, and no horizontal movement. The chewing simulator’s lower chambers received four specimens from each group at a time opposed by zirconia upper first molars mounted on the upper member of the chewing simulator in a class I relation angle’s classification. Each specimen endured 100,000 cycles, equivalent to six months of intraoral function (Fig. 3).

### Evaluation of wear resistance

After the specimens were detached from their holders, they were cleaned utilizing ultrasonic cleaning (UC-150, Sturdy Industrial, Taiwan) and subsequent air-drying to remove all debris. Then, each specimen was digitized with a desktop scanner (D850, 3Shape, Copenhagen, Denmark), and the STL file was saved to serve as measured data. For each specimen, the measured and reference data were input into surface matching software (Geomagic Control X; 3D Systems Inc.) to evaluate the volumetric occlusal wear of each specimen.

**Fig. 4:**
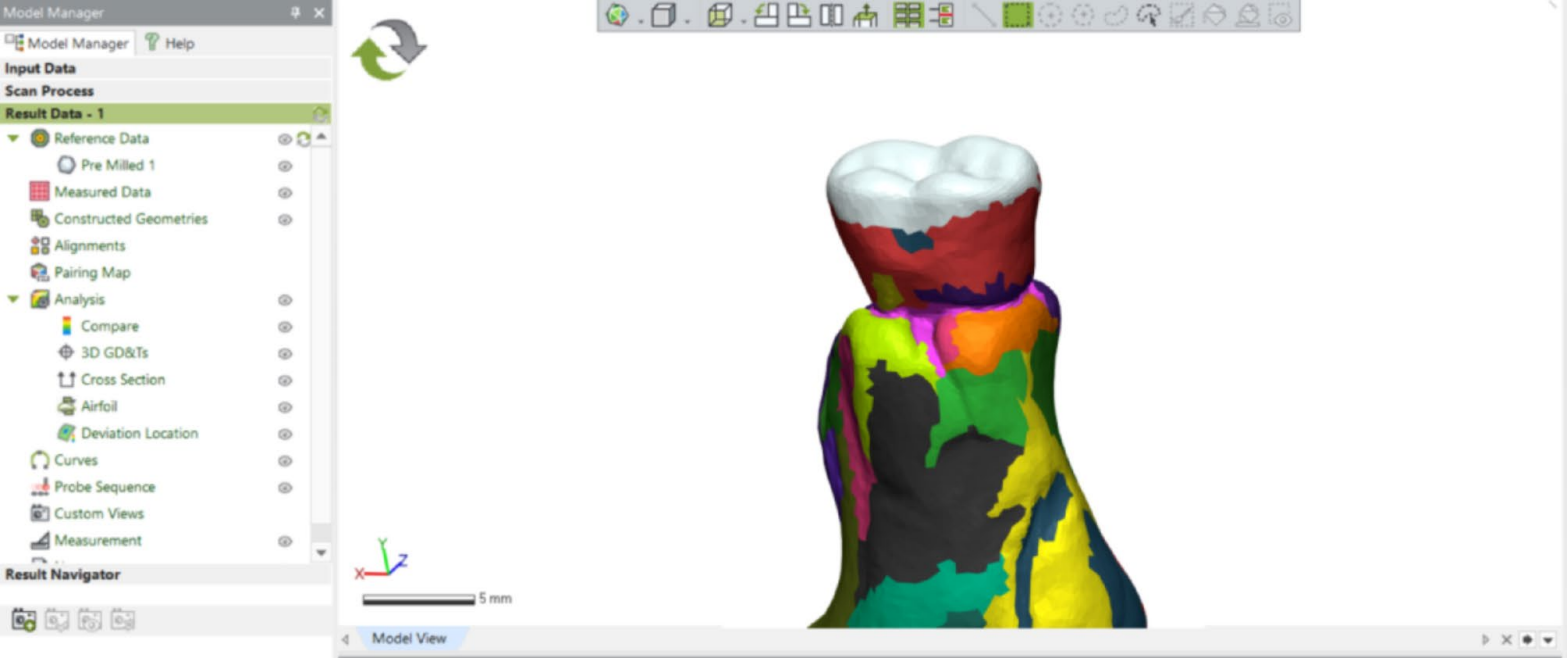
Occlusal surface segmentation

**Fig. 5:**
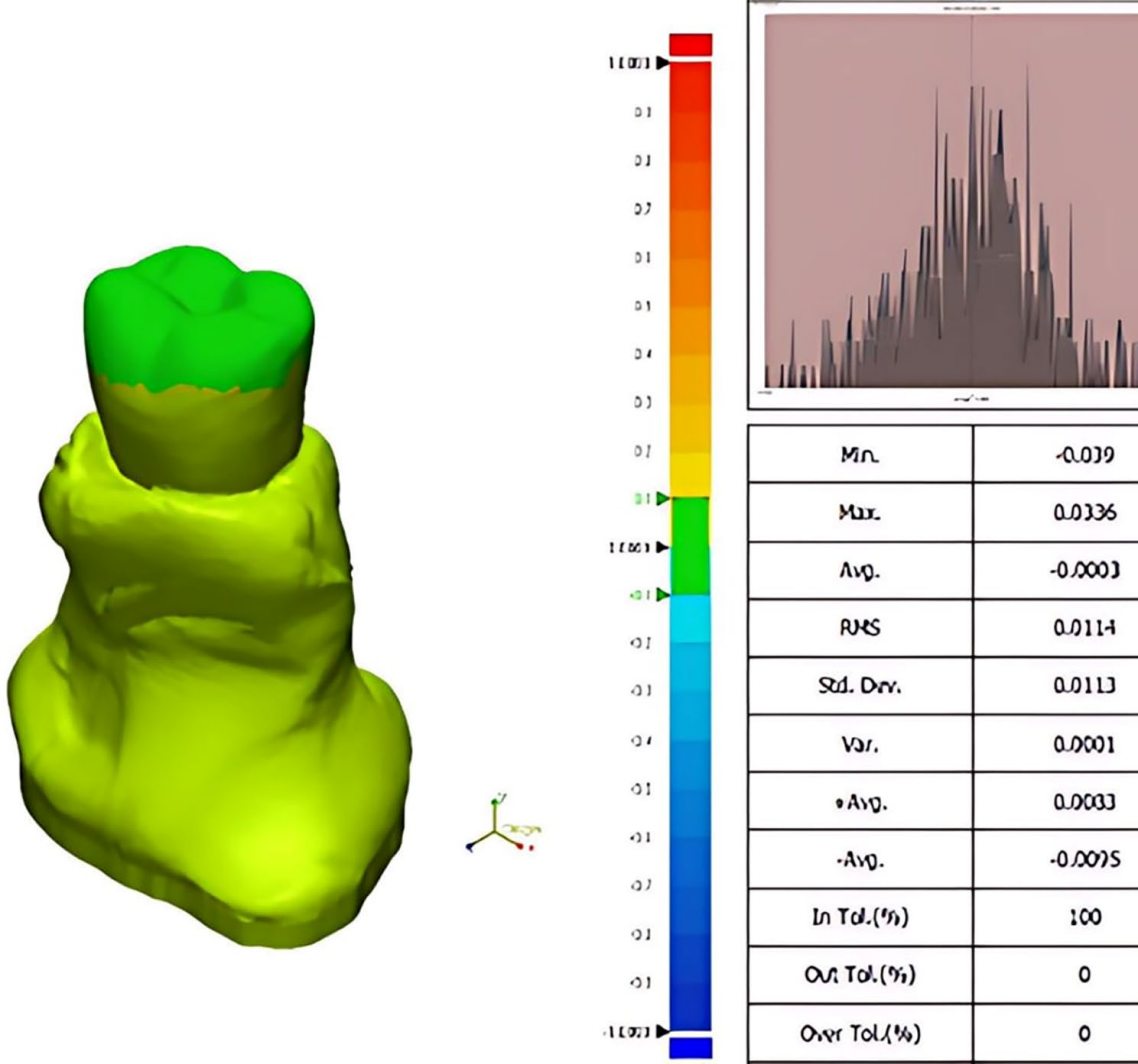
Colourimetric map of a milled tooth

Within the surface matching software, the occlusal surface of the reference data was segmented (Fig. 4) and designated as the comparison region. An initial alignment was performed, followed by a best-fit alignment for the reference data and the measured data [14]. To ensure accurate alignment, multiple cross-sectional views were generated. The 3D compare function was utilized with a defined colour bar range of 0.1 mm and a tolerance of 0.05 mm. A blue colour on a heat map scale represents areas of wear; the areas where the blue colour becomes darker indicate more significant volume loss (Fig. 5).

### Evaluation of colour stability

**Fig. 6:**
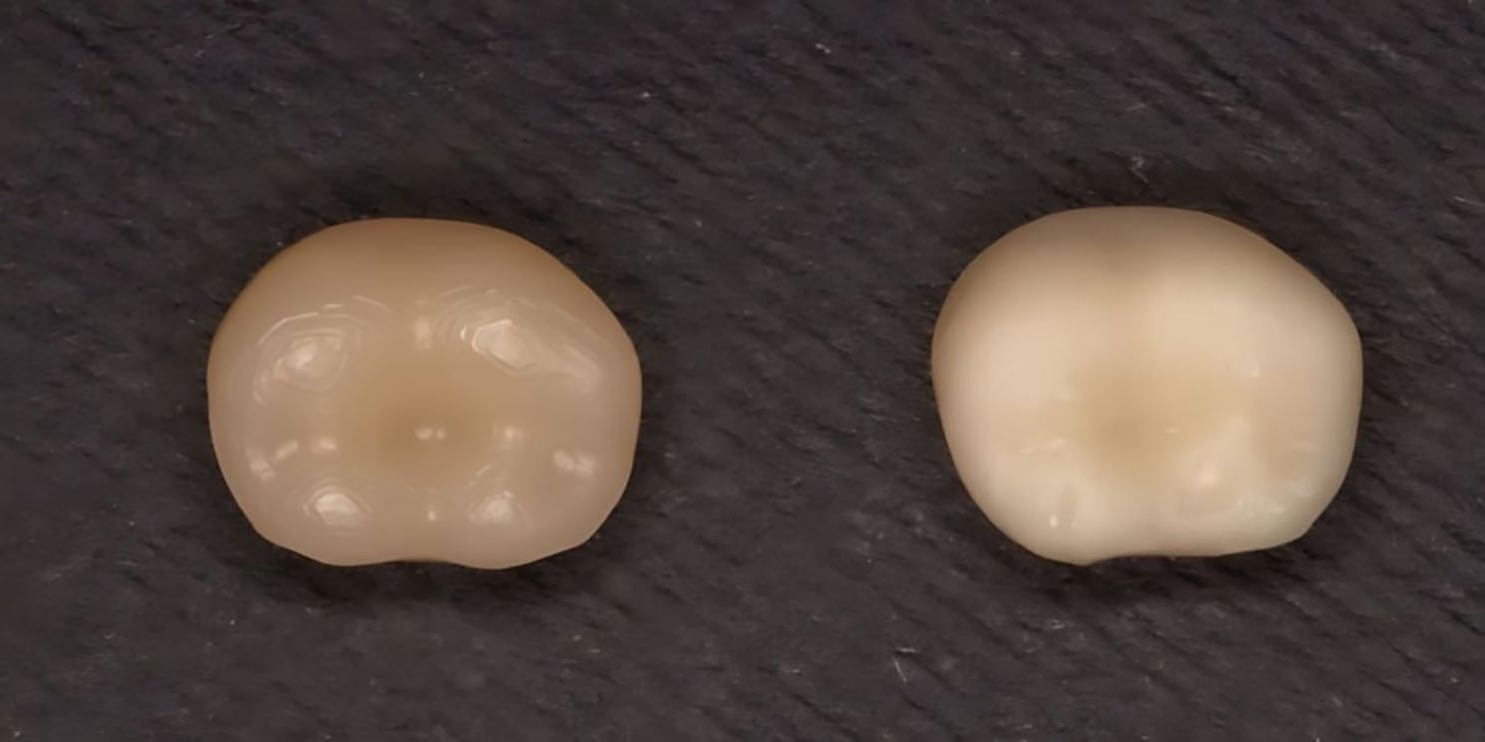
Denture teeth (milled tooth on the left, printed tooth on the right) on a black background for spectrophotometry

To evaluate the colour changes of the specimens, the 1976 Commission Internationale de l’Eclairage (CIE) L*a*b* colour space was used as recommended by the ISO/TR 28642:2016 report [15]. The colour changes were presented as Delta E (∆E_ab_ or ∆E_76_), where ∆E∗_ab_ = √ (∆L) 2 + (∆a) 2 + (∆b) 2. To start the assessment process, the specimens were placed on a black background (Fig. 6) [10]. Then, the spectrophotometer was calibrated according to the manufacturer’s recommendations prior to the measurement of each specimen to ensure accuracy and consistency of the colour assessments. Three records were made for each specimen utilizing a digital spectrophotometer (VITA Easyshade V, Germany). The average of the three records was then used as baseline reference data (L_b_, a_b_, b_b)_ [16].

The specimens were subjected to the thermocycler (Thermo Fisher Scientific ARCTIC A10B Refrigerated Circulator 1524101, REUZEit, Netherlands, European Union) with a regimen of 3500 cycles with temperatures between 278.15 and 328.15 K in each bath for 30 s, and a 10-second transmission interval between baths, following the protocol suggested by the ISO/TR 11405:1994 report [17]. Following cleaning with distilled water and drying using sterile gauze, a second spectrophotometric measurement was obtained. The final values (L_f_, a_f_, b_f_) were recorded under the same conditions as the baseline data by the same operator.

### Statistical analysis

Statistical analysis was conducted using SPSS 23.0 (Statistical Package for Scientific Studies, SPSS, Inc., Chicago, IL, USA) for Windows. Data were presented as mean ± range. The Kolmogorov-Smirnov test confirmed normality. One-way ANOVA followed by Tukey’s HSD post-hoc tests were used to compare groups. Statistical significance was set at *p* ≤ 0.05.

## Results

**Fig. 7:**
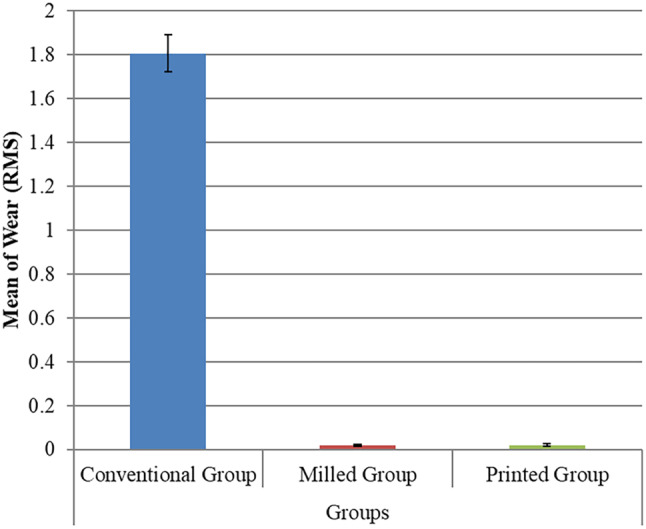
Bar chart represents mean and standard deviation values for the comparison between different groups regarding wear (RMS)

On comparing the volumetric changes, the ANOVA test showed statistically significant variations (*p* = 0.001) between the groups, with the highest mean value (RMS) in the conventional group 1.806 ± 0.085, followed by the printed group 0.021 ± 0.006, and then the milled group 0.019 ± 0.005, with no significant difference between the milled and printed groups as revealed by Tukey’s post hoc test (Table 1; Fig. 7).

**Table 1:** Intergroup comparison regarding wear (RMS)

Groups	N	Mean	±SD	±SE	95% C.I. for Mean		Min.	Max.	F-test	p-value
					Lower	Upper				
Conventional Group	7	1.806^A^	0.085	0.032	1.773	1.838	1.697	1.956	297.22	0.001**
Milled Group	7	0.019^B^	0.005	0.002	0.017	0.021	0.011	0.026		
Printed Group	7	0.021^B^	0.006	0.002	0.019	0.023	0.010	0.040		

SD = Standard deviation

SE = Standard Error

Min.= Minimum; Max.= Maximum

C.I.= Confidence Interval

*: Significance level at *P* ≤ 0.05

Different superscript letters in the same column indicate statistically significant difference between groups

**Fig. 8:**
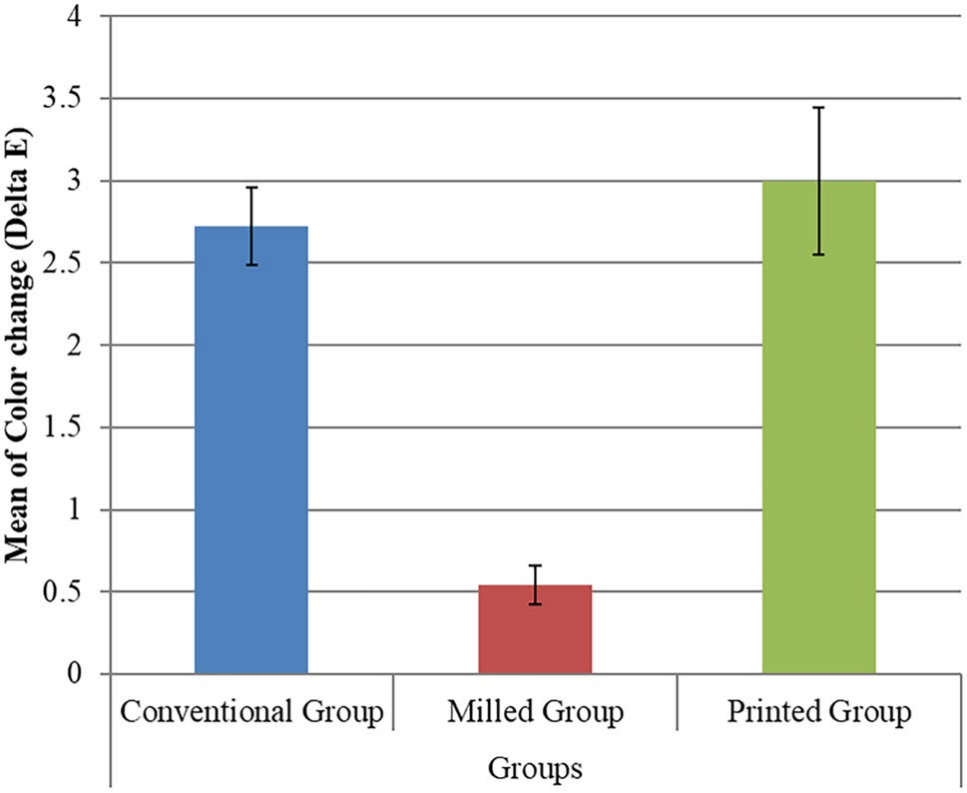
Bar chart represents mean and standard deviation values for the comparison between different groups regarding colour change (Delta E)

Regarding the intergroup comparison for the colour change, the highest mean value of colour change (Delta E) was recorded in the printed group 2.996 ± 0.445, followed by the conventional group 2.725 ± 0.234, and then the milled group 0.539 ± 0.118. The ANOVA test demonstrated a statistically significant difference between groups (*P* = 0.001). Tukey’s post hoc test revealed no significant difference between the Conventional group and the Printed group (Table 2; Fig. 8).

**Table 2:** Intergroup comparison of colour change (Delta E)

Groups	N	Mean	±SD	±SE	95% C.I. for Mean		Min.	Max.	F-test	p-value
					Lower	Upper				
Conventional Group	7	2.725^A^	0.234	0.088	2.637	2.814	2.391	3.011	133.81	0.001**
Milled Group	7	0.539^B^	0.118	0.045	0.494	0.584	0.258	0.763		
Printed Group	7	2.996^A^	0.445	0.168	2.828	3.164	2.510	3.659		

SD = Standard deviation

SE = Standard Error

Min.= Minimum; Max.= Maximum

C.I.= Confidence Interval

*: Significance level at *P* ≤ 0.05

Different superscript letters in the same column indicate statistically significant difference between groups

## Discussion

The null hypothesis of no statistically significant difference in the wear resistance and colour stability between prefabricated, milled, and 3D-printed teeth was rejected.

Prefabricated teeth are considered the criterion standard, so they were used in the first group, which is considered the control group [11]. It has been reported that pre-polymerized PMMA blanks used for milling denture teeth offer improved dimensional stability and mechanical properties compared to conventional resin, so they were presented in the second group [18, 19]. Dry milling was selected to simplify the procedures since it requires less time and fewer cutting forces, increasing the tool life while giving superior surface qualities [3]. Due to its versatility, 3D printing technology was selected for denture tooth manufacturing as it effectively addresses the limitations of the milling technique, including the availability of a wide range of printing machines, the minimal waste of raw material, and the capability to produce complex geometries. These advantages make it a key technology for future digital dentistry, but further studies are needed for the evaluation of the mechanical and biological properties of the printed prosthesis [6]. The 3D printing resin used for printing the denture teeth of the third group has been the most commonly used among the previous studies because of its good mechanical properties [19, 20].

In wear evaluation studies, the mandibular first molar is frequently used as a representative tooth because its selection often aims to standardise samples for comparison across different materials or testing conditions. As a posterior tooth, the first molar is subjected to increased occlusal loads during chewing compared to anterior teeth, making it a critical site for assessing wear [21]. For reproducible wear evaluation, a dental surveyor (Ney surveyor) was used to ensure that the loading surface of artificial teeth was parallel during the wear test, which is essential for consistent force distribution during wear simulation. This meticulous alignment helps to minimise variability in the wear process that might arise from inconsistent contact [12].

In the 3D printing group, the supporting arms were added on the ridge lab portion of the teeth to maintain the intactness of the occlusal surface and allow for better wear resistance evaluation [22, 23]. The accuracy of 3D-printed objects is affected by the type of printer used. DLP printer, which cures the resin layers using a light projector, was used as it offers high accuracy and precision [24].

After manufacturing the specimens, each was numbered to allow for accurate superimposition of the STL files for wear evaluation. A desktop scanner (3Shape D850) was used to scan each specimen as it has an accuracy of 7–8 μm [25].

Tooth surface wear can be evaluated using either two-body or three-body wear methods. Previous research [26] has focused on two-body wear, which models the direct contact between opposing tooth surfaces. In bilaterally balanced complete dentures, two-body wear is primarily observed during parafunctional activities like bruxism and swallowing. Consequently, the two-body wear method was selected for this study.

In this study, a programmable controlled chewing simulator was used. It simulates a two-body wear test where each acrylic tooth sample was in direct contact with a zirconia tooth antagonist in the presence of artificial saliva to wash any debris formed regularly to eliminate the possibility of a three-body wear test. This methodology is an attempt to simulate clinical situations [12]. As reported by Bonda A. et al. [27], the glandosane’s formula used as an artificial saliva presents a dynamic viscosity comparable to the viscosity of human saliva. To assess the denture teeth’ wear resistance, different materials have been used as antagonists, including zirconia, acrylic resin, steatite, stainless steel, and human enamel [26]. A maxillary first molar made from monolithic zirconia was used as an antagonist because of the mechanical properties that allow it to retain its shape during the chewing simulation process, ensuring consistent results [28]. A significant limitation of using natural human enamel as an antagonist is the impossibility of standardising its composition, structure, and therefore, its wear behaviour [26]. Enamel properties can exhibit high inter- and intraindividual variations [29].

Three-dimensional scanning and matching software is the preferred method for measuring wear, as stated by Wulfman et al. [9]. Unlike many methods that rely on comparing sequential 3D images, this study required only a single post-wear scan. Geomagic Control X software was employed for wear assessment. Surface matching and best-fit algorithms were used to superimpose post-wear scans onto initial scans, enabling precise digital measurements that surpass the accuracy of traditional physical methods [9]. Geomagic Control X offers unparalleled ease of use and intuitive comprehension for 3D inspection in any manufacturing workflow [30, 31].

The highest mean wear (RMS) value was reported in the conventional group, followed by the printed group and then the milled group. This coincides with what was reported by Myagmar G. et al. [32] when comparing the wear volume loss of conventional, 3D-printed, and milled resin. Myagmar G. et al. [32] reported that conventional resin has a higher wear volume loss than 3D-printed and milled resin. The same results were reported by Rayyan MM et al. [33] and Stawarczyk B et al. [34] on comparing the CAD/CAM milled resin with the conventional resin. They justified their results by the fact that pre-polymerized resin used in the fabrication of milled teeth has higher mechanical properties compared with conventional resin. Further supporting these findings, Pham et al. [11] stated that the wear resistance of denture teeth is significantly affected by their material. They justified that the 3D-printed teeth have higher wear resistance when compared with three types of prefabricated teeth where a monolithic zirconia stylus was used as an antagonist. They attributed their results to the uniform and homogenous composition of the 3D-printed teeth, as they are composed of a single resin material, unlike the prefabricated teeth, which consist of multiple layers that vary in their chemical composition [11, 12].

Enhanced wear resistance can lead to the improved longevity of dentures, as the teeth will be less prone to material loss during mastication and parafunctional activities. This is crucial because excessive wear can result in the loss of the vertical dimension of occlusion (VDO), which can negatively affect chewing efficiency, aesthetics, and potentially lead to mechanical failures of the prosthesis [11]. By maintaining a more stable occlusal relationship over time, milled PMMA teeth could contribute to better masticatory function and patient comfort, potentially reducing the need for frequent denture adjustments or replacements due to wear [21].

Given that milled PMMA generally exhibits improved mechanical properties compared to conventional PMMA [35], it may be a preferred material in high-stress scenarios where increased strength and durability are required, such as in implant-supported prostheses or for patients with parafunctional habits. The industrial polymerization process for milled resins can lead to a higher degree of polymerization, fewer pores, and minimal residual monomer content, potentially contributing to better resistance to stress and wear compared to conventional PMMA [32].

Tooth colour assessment can be performed either by using subjective visual methods, such as comparing to an acrylic resin shade guide, or objective instrumental methods, such as spectrophotometry. However, spectrophotometry provides a more comprehensive colour assessment over the use of the acrylic resin shade guide by generating numerical values for various colour coordinates [36]. As stated in Tieh et al.’s [10] systematic review, numerous studies used spectrophotometers for colour stability assessment, with the Vita Easy Shade being the most widely used model.

The 3D-printed teeth were positioned in a post-curing unit for 15 min. This can be justified as the post-processing methods, particularly post-curing, play a significant role in enhancing the colour stability of 3D-printed resins. Studies have shown that extending the post-curing time can lead to a reduction in the stainability of these materials [37, 38]. This improvement is often attributed to a higher degree of conversion achieved during prolonged post-curing, which results in fewer unreacted monomers and photo-initiators [39].

Aging of the specimens was conducted in a thermocycler, with a regimen of 3500 cycles with temperatures between 278.15 and 328.15 K for 30 s, and a transmission time of 10 s between each bath as the ISO/TR 11405:1994 report recommended [17]. Previous studies reported this regimen [16, 40, 41].

For colour readouts, the test specimens were placed on a standard, often-used black background following the ISO/TR Z8642:Z016 [15] to assess the translucency parameter (TP). The TP is calculated by measuring the colour difference of the same specimen over a black background. This method helps determine how much light passes through the material, which is crucial for understanding its translucency [10].

The VITA Easyshade spectrophotometer was selected for colour assessment in this study as it has been reported by Tieh et al. [42] to be highly reliable (96.4%) and accurate (92.6%). It incorporates an embedded fiber optic light for accurate tooth shade measurement in any lighting environment.

The Vita Easyshade spectrophotometer determined the colour change using the CIE L*a*b* system. It consists of three coordinates: L* represents luminosity, while a* and b* represent the dimension of tonality or colour. Instead of comparing the three colour coordinates (L*, a*, and b*), a ΔE formula is suggested to evaluate colour change, where ∆E∗ab = √ (∆L) 2 + (∆a) 2 + (∆b) 2. This formula, derived from CIE 1971, provides a more comprehensive assessment by summarizing the combined effect of changes in L*, a*, and b* on the perceived colour [43, 44].

The highest mean value of colour change (ΔE) was reported in the printed group, followed by the conventional group, and then the milled group. This can be owed to the increased water sorption of 3D-printed resin causing higher pigment penetration and resin deterioration, as justified by Gruber et al. [40] and Tieh et al. [42]. Gruber et al. [40] reported that 3D-printed denture resins showed the greatest colour change compared to conventional heat-cured resins and CAD/CAM subtractively manufactured denture resins. Berli et al. [18] reported that 3D-printed resins showed significantly higher water sorption post-thermal cycling. Furthermore, the study highlighted that water absorption significantly reduced flexural strength and accelerated the degradation of these 3D-printed resins.

Kamal et al. [45] compared the colour stability of three different CAD/CAM milled denture materials: polyether ether ketone (PEEK), acetal resin, and acrylic resin, and found that acrylic resin exhibited the highest statistically significant colour change, followed by PEEK, while acetal resin showed the least and justified that the acrylic resin had the highest colour change caused by water sorption and surface porosity. Alp et al. [46] evaluated the colour stability of conventional and different pre-polymerized CAD-CAM PMMA denture materials. They concluded that pre-polymerized resin showed the least colour change because of the low water absorption properties of pre-polymerized PMMA denture materials, as stated by their manufacturers, and the denture base materials’ hydrophilic properties.

Also, another justification for the lowered printed teeth colour stability by Arora et al. [36] is that a potential cause of surface deterioration in 3D-printed parts could be the resin’s filler content. Resins with lower filler content, a common characteristic of 3D-printing materials, tend to exhibit increased surface wear and tear. This is because fillers enhance the resin’s durability. While low filler content aids in achieving smooth prints and maintaining low viscosity for 3D printing, it can compromise the material’s resistance to wear. Furthermore, filler particle sedimentation during storage can result in uneven resin layers during the printing process. This inconsistency can disrupt the polymerization process, further contributing to surface degradation. An additional influence on surface deterioration is the presence of residual monomers within the resin. High residual monomer levels can lead to water absorption and subsequent material expansion. This dimensional instability can degrade the surface and compromise the overall mechanical properties of the 3D-printed part.

Different 3D-printed resins display varying levels of water sorption primarily due to their inherent material composition, particularly the hydrophilic nature of the monomers used and the type and amount of cross-linking agents. The inclusion and bonding of filler particles can either decrease water uptake by reducing the resin matrix or increase it if poor bonding creates pathways [42]. Furthermore, the degree of polymerization achieved during printing and post-curing is critical, as lower conversion rates leave more susceptible residual monomers [40]. The layer-by-layer manufacturing process in 3D printing can also introduce areas of weakness between layers, facilitating water ingress, while surface porosity and roughness offer a larger interaction area for water absorption. Consequently, the specific formulation and processing of each unique 3D-printed resin significantly dictate its water sorption characteristics, as evidenced by studies showing considerable variability among different materials and manufacturers [18].

### Limitations and future scope

Firstly, the investigation was conducted in vitro, which may not precisely mimic the intraoral conditions. Secondly, the study focused on a limited number of CAD/CAM materials, and further research is warranted to compare the performance of different brands. Finally, clinical trials are necessary to confirm the findings of this study in vivo situations and assess the long-term clinical outcomes of these materials.

## Conclusion

Based on the results of this in vitro study, it was concluded that.

1. Milled PMMA generally demonstrates better wear resistance and colour stability compared to 3D-printed and conventionally processed PMMA within the confines of this in vitro investigation. Further clinical validation is required, though, to verify these conclusions.
2. 3D-printed PMMA exhibits comparable wear resistance to milled PMMA.
3. 3D-printed PMMA demonstrated comparable colour stability to conventional resin.

### Recommendations

Future research should focus on 3D-printing resin formulation modifications for improved colour stability. Future studies could increase the sample size to enhance statistical reliability.

Further investigations focusing on the long-term therapeutic effectiveness and clinical performance of milled and 3D-printed PMMA materials could provide a clearer path for future research while maintaining the clarity and conciseness of our findings.

## Data Availability

Available upon request from the corresponding author.

## Abbreviations

PMMA: Polymethyl methacrylate
CAD-CAM: Computer-aided design and computer-aided manufacturing
SLA: Stereolithography
STL: Standard Tessellation Language
CIE: Commission Internationale de l’Eclairage (International Commission on Illumination)
ΔE: Delta E
PEEK: Polyether ether ketone

## References

[1] Saeed F, Muhammad N, Khan AS, Sharif F, Rahim A, Ahmad P, et al. Prosthodontics dental materials: from conventional to unconventional. Mater Sci Eng C Mater Biol Appl. 2020;106:110167. 10.1016/j.msec.2019.110167. 31753414 10.1016/j.msec.2019.110167

[2] de Oliveira E, Zancanaro de Figueiredo E, Spohr AM, Lima Grossi M. Properties of acrylic resin for CAD/CAM: A systematic review and Meta-Analysis of in vitro studies. J Prosthodont. 2021;30(8):656–64. 10.1111/jopr.13394. 34036676 10.1111/jopr.13394

[3] Bae EJ, Jeong ID, Kim WC, Kim JH. A comparative study of additive and subtractive manufacturing for dental restorations. J Prosthet Dent. 2017;118(2):187–93. 10.1016/j.prosdent.2016.11.004. 28089336 10.1016/j.prosdent.2016.11.004

[4] Baba NZ, Goodacre BJ, Goodacre CJ, Muller F, Wagner S. CAD/CAM complete denture systems and physical properties: A review of the literature. J Prosthodont. 2021;30(S2):113–24. 10.1111/jopr.13243. 32844510 10.1111/jopr.13243

[5] Barazanchi A, Li KC, Al-Amleh B, Lyons K, Waddell JN. Additive technology: update on current materials and applications in dentistry. J Prosthodont. 2016;26(2):156–63. 10.1111/jopr.12510. 27662423 10.1111/jopr.12510

[6] Anadioti E, Musharbash L, Blatz MB, Papavasiliou G, Kamposiora P. 3D printed complete removable dental prostheses: a narrative review. BMC Oral Health. 2020;20(1):343. 10.1186/s12903-020-01328-8. 33246466 10.1186/s12903-020-01328-8 PMC7694312

[7] Eaton KA. The development of digital dentistry in the UK: an overview. Prim Dent J. 2022;11(4):94–8. 10.1177/20501684221134198. 36533365 10.1177/20501684221134198

[8] Beleidy M, Ziada A. Comparative wear analysis of conventional versus CAD/CAM composite veneered PEEK crowns using 3D surface deviation. Egypt Dent J. 2022;68(3):2721–31. 10.21608/edj.2022.139867.2116.

[9] Wulfman C, Koenig V, Mainjot AK. Wear measurement of dental tissues and materials in clinical studies: A systematic review. Dent Mater. 2018;34(6):825–50. 10.1016/j.dental.2018.03.002. 29627079 10.1016/j.dental.2018.03.002

[10] Tieh MT, Waddell JN, Choi JJE. Optical properties and color stability of denture Teeth-A systematic review. J Prosthodont. 2022;31(5):385–98. 10.1111/jopr.13429. 34516027 10.1111/jopr.13429

[11] Pham DM, Gonzalez MD, Ontiveros JC, Kasper FK, Frey GN, Belles DM. Wear resistance of 3D printed and prefabricated denture teeth opposing zirconia. J Prosthodont. 2021;30(9):804–10. 10.1111/jopr.13339. 33486808 10.1111/jopr.13339

[12] Saadi ASA, El-Damanhoury HM, Khalifa N. 2D and 3D wear analysis of 3D printed and prefabricated artificial teeth. Int Dent J. 2023;73(1):87–92. 10.1016/j.identj.2022.10.002. 36372591 10.1016/j.identj.2022.10.002 PMC9875236

[13] Stock V, Schmidlin PR, Merk S, Wagner C, Roos M, Eichberger M, et al. PEEK primary crowns with Cobalt-Chromium, zirconia and galvanic secondary crowns with different Tapers-A comparison of retention forces. Mater (Basel). 2016;9(3). 10.3390/ma9030187. 10.3390/ma9030187 PMC5456689 28773311

[14] Gad MM, Alalawi H, Akhtar S, Al-Ghamdi R, Alghamdi R, Al-Jefri A, et al. Strength and wear behavior of Three-Dimensional printed and prefabricated denture teeth: an in vitro comparative analysis. Eur J Dentistry. 2023;17(04):1248–56. 10.1055/s-0042-1759885. 10.1055/s-0042-1759885 PMC10756787 36669653

[15] ISO/TR-28642:2016(E). Dentistry — Guidance on colour measurement [Internet] 2016 Dec 01.

[16] Khomprang R, Sripetchdanond J, Chengprapakorn W. Effect of coffee thermocycling on color stability and translucency of CAD-CAM polychromatic high translucent zirconia compared with Lithium disilicate glass ceramic. Clin Exp Dent Res. 2024;10(4):e918. 10.1002/cre2.918. 38970231 10.1002/cre2.918 PMC11226548

[17] ISO/TR-11405. Dental materials— Guidance on testing of adhesion to tooth structure [Internet] 1994 Dec 15.

[18] Berli C, Thieringer FM, Sharma N, Muller JA, Dedem P, Fischer J, et al. Comparing the mechanical properties of pressed, milled, and 3D-printed resins for occlusal devices. J Prosthet Dent. 2020;124(6):780–6. 10.1016/j.prosdent.2019.10.024. 31955837 10.1016/j.prosdent.2019.10.024

[19] Veerapeindee P, Rungsiyakull P, Jia-Mahasap W. Wear resistance of 3D printed, milled, and prefabricated methacrylate-based resin materials: an in vitro study. J Prosthet Dent. 2024. 10.1016/j.prosdent.2024.12.006. 39709262 10.1016/j.prosdent.2024.12.006

[20] Farahat AMS, Refai OM, Elsherbeeny YS. Trueness of maxillary complete dentures duplicated by using conventional and 3D printing techniques: A comparative in vitro study. J Prosthet Dent. 2024. 10.1016/j.prosdent.2024.12.004. 39701842 10.1016/j.prosdent.2024.12.004

[21] Abbas M, Sakr H. Wear performance of Nano-Composite artificial denture teeth. Egypt Dent J. 2017;63(4):2535–44. 10.21608/edj.2017.76074.

[22] Gao H, Yang Z, Lin WS, Tan J, Chen L. The effect of build orientation on the dimensional accuracy of 3D-Printed mandibular complete dentures manufactured with a multijet 3D printer. J Prosthodont. 2021;30(8):684–9. 10.1111/jopr.13330. 33459450 10.1111/jopr.13330

[23] Unkovskiy A, Bui PH, Schille C, Geis-Gerstorfer J, Huettig F, Spintzyk S. Objects build orientation, positioning, and curing influence dimensional accuracy and flexural properties of stereolithographically printed resin. Dent Mater. 2018;34(12):e324–33. 10.1016/j.dental.2018.09.011. 30293688 10.1016/j.dental.2018.09.011

[24] Sim MY, Park JB, Kim DY, Kim HY, Park JM. Dimensional accuracy and surface characteristics of complete-arch cast manufactured by six 3D printers. Heliyon. 2024;10(10):e30996. 10.1016/j.heliyon.2024.e30996. 38778963 10.1016/j.heliyon.2024.e30996 PMC11109808

[25] Pilecco RO, Machry RV, Baldi A, Tribst JPM, Sarkis-Onofre R, Valandro LF, et al. Influence of CAD-CAM milling strategies on the outcome of indirect restorations: A scoping review. J Prosthet Dent. 2024;131(5):811e. 10.1016/j.prosdent.2024.02.021. 10.1016/j.prosdent.2024.02.021 38480018

[26] Cha HS, Park JM, Kim TH, Lee JH. Wear resistance of 3D-printed denture tooth resin opposing zirconia and metal antagonists. J Prosthet Dent. 2020;124(3):387–94. 10.1016/j.prosdent.2019.09.004. 31784192 10.1016/j.prosdent.2019.09.004

[27] Foglio-Bonda A, Foglio-Bonda PL, Bottini M, Pezzotti F, Migliario M. Chemical-physical characteristics of artificial saliva substitutes: rheological evaluation. Eur Rev Med Pharmacol Sci. 2022;26(21):7833–9. 10.26355/eurrev_202211_30132. 36394731 10.26355/eurrev_202211_30132

[28] Kim ST, Cook DR, Albouy JP, De Kok I, Sulaiman TA. Linear and volumetric wear of conventional and milled denture teeth. J Esthet Restor Dent. 2022;34(3):519–26. 10.1111/jerd.12868. 35019205 10.1111/jerd.12868

[29] Alshehri A. The wear of acrylic resin and composite resin teeth against polished zirconia. 2018.

[30] Goodacre BJ, Goodacre CJ, Baba NZ, Kattadiyil MT. Comparison of denture base adaptation between CAD-CAM and conventional fabrication techniques. J Prosthet Dent. 2016;116(2):249–56. 10.1016/j.prosdent.2016.02.017. 27112416 10.1016/j.prosdent.2016.02.017

[31] Wang C, Shi YF, Xie PJ, Wu JH. Accuracy of digital complete dentures: A systematic review of in vitro studies. J Prosthet Dent. 2021;125(2):249–56. 10.1016/j.prosdent.2020.01.004. 32115218 10.1016/j.prosdent.2020.01.004

[32] Myagmar G, Lee JH, Ahn JS, Yeo IL, Yoon HI, Han JS. Wear of 3D printed and CAD/CAM milled interim resin materials after chewing simulation. J Adv Prosthodont. 2021;13(3):144–51. 10.4047/jap.2021.13.3.144. 34234924 10.4047/jap.2021.13.3.144 PMC8250192

[33] Rayyan MM, Aboushelib M, Sayed NM, Ibrahim A, Jimbo R. Comparison of interim restorations fabricated by CAD/CAM with those fabricated manually. J Prosthet Dent. 2015;114(3):414–9. 10.1016/j.prosdent.2015.03.007. 26001490 10.1016/j.prosdent.2015.03.007

[34] Stawarczyk B, Ozcan M, Trottmann A, Schmutz F, Roos M, Hammerle C. Two-body wear rate of CAD/CAM resin blocks and their enamel antagonists. J Prosthet Dent. 2013;109(5):325–32. 10.1016/S0022-3913(13)60309-1. 23684283 10.1016/S0022-3913(13)60309-1

[35] Esquivel J, Lawson NC, Kee E, Bruggers K, Blatz MB. Wear of resin teeth opposing zirconia. J Prosthet Dent. 2020;124(4):488–93. 10.1016/j.prosdent.2019.11.004. 31952860 10.1016/j.prosdent.2019.11.004

[36] Arora O, Ahmed N, Siurkel Y, Ronsivalle V, Cicciu M, Minervini G. A comparative evaluation of physical properties of CAD/CAM complete denture resins- an in vitro study. BMC Oral Health. 2024;24(1):65. 10.1186/s12903-023-03708-2. 38200506 10.1186/s12903-023-03708-2 PMC10777544

[37] Dizon JRC, Gache CCL, Cascolan HMS, Cancino LT, Advincula RC. Post-Processing of 3D-Printed polymers. Technologies. 2021;9(3). 10.3390/technologies9030061.

[38] Baytur S, Diken Turksayar AA. Effects of post-polymerization conditions on color properties, surface roughness, and flexural strength of 3D-printed permanent resin material after thermal aging. J Prosthodont. 2025;34(3):298–307. 10.1111/jopr.13818. 38102064 10.1111/jopr.13818 PMC11880970

[39] Hassanpour M, Narongdej P, Alterman N, Moghtadernejad S, Barjasteh E. Effects of Post-Processing parameters on 3D-Printed dental appliances: A review. Polym (Basel). 2024;16(19). 10.3390/polym16192795. 10.3390/polym16192795 PMC11479229 39408505

[40] Gruber S, Kamnoedboon P, Ozcan M, Srinivasan M. CAD/CAM complete denture resins: an in vitro evaluation of color stability. J Prosthodont. 2021;30(5):430–9. 10.1111/jopr.13246. 32864812 10.1111/jopr.13246

[41] Sahin O, Dede DO, Koroglu A, Yilmaz B. Influence of surface sealant agents on the surface roughness and color stability of artificial teeth. J Prosthet Dent. 2015;114(1):130–7. 10.1016/j.prosdent.2015.02.009. 25913372 10.1016/j.prosdent.2015.02.009

[42] Tieh MT, Waddell JN, Choi JJE. Optical and mechanical properties of conventional, milled and 3D-printed denture teeth. J Mech Behav Biomed Mater. 2022;126:105061. 10.1016/j.jmbbm.2021.105061. 34963102 10.1016/j.jmbbm.2021.105061

[43] Luo MR, Cui G, Rigg B. The development of the CIE 2000 colour-difference formula: CIEDE2000. Color Res Application. 2001;26(5):340–50. 10.1002/col.1049.

[44] Paravina RD, Ghinea R, Herrera LJ, Bona AD, Igiel C, Linninger M, et al. Color difference thresholds in dentistry. J Esthet Restor Dent. 2015;27(Suppl 1):S1–9. 10.1111/jerd.12149. 25886208 10.1111/jerd.12149

[45] Kamal MNM. Comparative evaluation of color stability between three different CAD/CAM milled denture base materials: an in vitro study. J Int Dent Med Res. 2020;13(3).

[46] Alp G, Johnston WM, Yilmaz B. Optical properties and surface roughness of prepolymerized poly(methyl methacrylate) denture base materials. J Prosthet Dent. 2019;121(2):347–52. 10.1016/j.prosdent.2018.03.001. 30143239 10.1016/j.prosdent.2018.03.001

